# Genome Sequences of Two Morphologically Distinct and Thermophilic *Bacillus coagulans* Strains, H-1 and XZL9

**DOI:** 10.1128/genomeA.00254-13

**Published:** 2013-05-16

**Authors:** Ke Xu, Fei Su, Fei Tao, Chao Li, Jun Ni, Ping Xu

**Affiliations:** State Key Laboratory of Microbial Metabolism and School of Life Sciences and Biotechnology, Shanghai Jiao Tong University, Shanghai, China

## Abstract

Two thermophilic *Bacillus coagulans* strains, H-1 and XZL9, both of which were isolated from soils, have different morphological properties. Strain XZL9 but not H-1 is an efficient pentose-utilizing producer of important platform compounds, such as l-lactic acid and 2,3-butanediol. Here we announce the 2.86- and 3.43-Mb sequences of their genomes.

## GENOME ANNOUNCEMENT

Many strains present morphological, cytological, and physiological alterations under different environments. Morphological features of microbial cells may play a role in fermentations (1–3). In previous studies, *Bacillus coagulans* was proposed to be a potential good producer for platform chemicals, such as l-lactic acid, 2,3-butanediol, and ethanol (4–9). Both of the *B. coagulans* strains H-1 (CCTCC M 2013105) and XZL9 (DSM 23184) are good l-lactic acid producers. These two strains were isolated by our group from soils and were found to share many good characteristics, such as high optical purity, nonsterilization fermentation under high temperatures, and high productivity. However, a morphological change of strain H-1, which is unable to utilize pentose, transforming it from a filamentous to a rod shape, is accompanied by reduced production rates and even growth limits. Meanwhile, the stable morphology of strain XZL9 can produce high l-lactic acid (130 g/liter) from xylose, with a high optical purity of 99% and a high yield of 98% at a temperature of 50°C (data not shown). To discriminate the relationship between physicochemical and metabolic properties, we determined the whole-genome sequences of strains H-1 and XZL9.

We obtained the raw data sets of strains H-1 and XZL9 using the Illumina HiSeq 2000 system. A total of 9,915,274 filtered reads for strain H-1 were assembled into 337 contigs, and a total of 15,532,424 filtered reads for strain XZL9 were assembled into 45 contigs using VELVET (10). The genome annotations were performed by the RAST server (11). The functional descriptions were determined using Clusters of Orthologous Genes (12) and KEGG (13). The genes encoding tRNAs and rRNAs were identified by tRNAscan-SE (14) and RNAmmer (15), respectively.

The draft genome sequences of strains H-1 and XZL9 consist of 2,862,880 bases and 3,426,041 bases, with GC contents of 47.3% and 46.5%, respectively. There are 104 and 101 predicted RNAs in strains H-1 and XZL9, respectively. A total of 329 subsystems for strain H-1 and 531 subsystems for strain XZL9 were identified by RAST. In the results of RAST, we did not find significant differences in the genes involved in cell division of the two strains. Both of them possess the key structure of a cytokinetic ring, including *ftsAKLIWZ* and missing the essential gene *zipA*, which connects the ring with the cell membrane. This implies that *B. coagulans* has a common cell division process similar to that of typical rod-shaped cells, such as *Escherichia coli* and *Bacillus subtilis*. Besides these, in the subsystem of the cell wall and capsule, there are 74 genes and 108 genes for strains H-1 and XZL9, respectively. We have carefully compared the two strains, finding that the systems of rhamnose biosynthesis and sialic acid metabolism were completely absent in strain H-1. This might lead to the unstable morphology of strain H-1, which needs to be determined by further experiments.

### Nucleotide sequence accession numbers.

The whole-genome shotgun project has been deposited at DDBJ/EMBL/GenBank under the accession numbers ANAQ00000000 for strain H-1 and ANAP00000000 for strain XZL9. The versions described in this paper are the first version, ANAQ01000000 and ANAP01000000.
